# Lung cancer screening in Finland: a prospective randomized trial

**DOI:** 10.2340/1651-226X.2025.43093

**Published:** 2025-06-11

**Authors:** Viktor Wichmann, Sanna Iivanainen, Lauri Mattila, Veli-Pekka Kokkonen, Airi Jartti, Antti Kurtti, Riitta Kaarteenaho, Heidi Andersen, Antti Jekunen, Tuula Vasankari, Jussi Koivunen

**Affiliations:** a Cancer Center Oulu University Hospital, Oulu, Finland; b University of Oulu, Oulu, Finland; c Medical Research Center Oulu, Oulu, Finland; d Department of Diagnostic Radiology, Oulu University Hospital, Oulu, Finland; e Center of Internal Medicine and Respiratory Medicine, Oulu University Hospital, Oulu, Finland; f Research Unit of Biomedicine and Internal Medicine, University of Oulu, Oulu, Finland; g Vaasa Central Hospital, Finland; h University of Turku, Finland; i Filha, Helsinki, Finland

**Keywords:** Lung cancer, screening, LDCT, smoking cessation

## Abstract

**Background:**

Early detection of lung cancer with low-dose computed tomography (LDCT) screening can shift diagnoses to early-stage disease and improve survival. However, LDCT has several challenges such as high false positive rate and indefinite cost-effectiveness. We report here secondary and exploratory endpoints of the Low-dose CT screening for lung cancer combined with different smoking cessation approach in Finland (LDCT-SC-FI) study including recruitment channels, LDCT performance, and long-term smoking cessation.

**Methods:**

In this study, we randomized 200 current smokers with a significant smoking history in 1:1 fashion to receive a smartphone application or standard of care written materials, both for smoking cessation. All underwent LDCT screening at baseline and at 1-year. Participants were recruited through multiple channels, including newspapers, internet advertisements, and healthcare referrals.

**Results:**

Newspaper advertisements were the most effective recruitment method, accounting for 74.5% of participants while minority came through referrals (2.5%). LDCT screening demonstrated uptake of 96.7% for both rounds combined. Six lung cancers were detected with a positive predictive value of 75%. Of the detected lung cancers, five were at stage I and all of these underwent curative intent treatment. Smoking cessation rates at 1-year were higher in the application (18.3%) than in the control arm (12.8%), though the difference was not statistically significant (odds ratio [OR]: 1.53, 95% confidence interval [CI]: 0.69–3.41).

**Interpretation:**

This study suggests that LDCT screening for lung cancer is feasible in Finland. The screening examination uptake was high with both screening rounds, while the positive predictive value for lung cancer detection remained at good level.

## Introduction

Lung cancer remains the leading cause of cancer-related deaths in Western countries, with smoking being the predominant risk factor [1]. Early-stage lung cancer is often asymptomatic, leading to diagnoses at advanced stages when curative intent treatments are not feasible. Lung cancer screening of individuals with marked smoking history using low-dose computed tomography (LDCT) has been shown to shift diagnoses toward earlier, localized stages of the disease, to decrease lung cancer mortality, and, possibly, to increase overall survival [2, 3]. United States Preventive Services Taskforce recommends annual LDCT for adults aged 50–80 years who have a 20 pack-year smoking history, currently smoke, or have quit within the past 15 years [4]. Elsewhere, LDCT screening has been integrated into national healthcare systems in only a few countries. In 2022, EU has recommended that the member states should explore the feasibility and effectiveness of lung cancer LDCT screening, for instance by implementation studies (EU council recommendation 2022/C 473/01). LDCT screening is currently not part of the national cancer screening program in Finland as in other Nordic countries.

Lung cancer screening has pitfalls, which makes its adoption challenging to cancer screening programs. Current protocols are based on age and smoking background of which the latter is generally not available in national registries or patient records. Therefore, participant invitations need to rely on methods such as voluntary enrollment or sending out a large quantity of invitations to a specific age group in which a minority is eligible for screening. LDCT as screening method provides a high rate of false positive findings, which result in additional workup and, possibly, anxiety. Furthermore, participation to post-baseline LDCT screens might decline and decrease the effectiveness of lung cancer screening [5, 6].

Integrating smoking cessation intervention is recommended within the LDCT screening program for lung cancer, as smoking cessation support is more effective when provided alongside cancer screening regardless of the screening result [7–10]. A systematic review has shown that 7–23% of the individuals participating in LDCT programs are successful in quitting smoking [11]. Nonetheless, the methods for smoking cessation in LDCT screening context are not well established.

We have initiated the first pilot of LDCT screening for lung cancer in Finland (LDCT-SC-FI). In this study, we investigated variable smoking cessation methods in a randomized controlled fashion and were able to show that the developed smartphone application increases the chance for smoking cessation by three-fold at 3 and 6 months [12]. We report here some of the secondary and exploratory endpoints of the LDCT-SC-FI study focusing on effectiveness of different recruitment channels, performance measures of LDCT, and long-term smoking cessation results. In brief, results of LDCT-SC-FI study suggest that lung screening with LDCT is feasible in Finland.

## Methods

### Study participants

The main inclusion criteria comprised an age of 50–74, a marked smoking history (smoked ≥ 15 cigarettes/day for ≥ 25 years or smoked ≥ 10 cigarettes/day for ≥ 30 years), an active smoking status (smoking during the last 2 weeks including regular [daily smoking] and occasional [non-daily smoking] habits), and access to a smartphone (iPhone or Android). The main exclusion criteria, as in the NELSON trial included a moderate or bad self-reported health (inability to climb two flights of stairs), current or past melanoma, lung, renal or breast cancer, and a chest CT examination less than 1 year before the inclusion [2]. Pre-screening was done by phone by a study nurse. Physical screening visit was performed at the site where participating individuals signed the informed consent. Eligibility was verified by a study nurse according to a checklist. We enrolled 201 participants fulfilling these criteria while the final analyses included 200 patients since one randomized individual dropped out before the first LDCT scan and was replaced. Participants were stratified by age and pack-years and randomized in 1:1 fashion to the developed smartphone application (experimental arm) or written material (standard of care). All the subjects were offered LDCT screening at baseline and at 1 year. Further methodology concerning the smartphone application used in the study is described in our previous article [12].

### Recruitment

The study recruitment was initiated on Nov 18th 2022 and the last subject was included Apr 14th 2023. Even though the study was planned to be a multicenter trial, the inclusion took place only in a single center (Oulu University Hospital, Oulu, Finland) because of the rapid inclusion at the first opened site. The recruitment was carried out by newspaper, internet advertisements and informing relevant healthcare units at hospital district.

### Imaging protocol and nodule management

The LDCT–SC–FI study protocol for LDCT interpretation followed the NELSON study protocol [2]. Low-dose CT scans without contrast media were acquired with the use of 128- or 384- multidetector CT scanners. All scans were evaluated individually by three radiologists specialized in thoracic radiology (LM, V-PK, and AiJa). Lesion volumes were assessed with the use of semiautomated software (Siemens Syngo. via MM Oncology). Identified lesions were classified into nodule category based on size (NODCAT) or volume doubling time (GROWCAT) [13]. Study subjects underwent LDCT screening within 6 weeks from randomization and were informed of the results by mail. If no further procedures were required (negative or NODCAT I-II), the next LDCT was scheduled for 1 years (±2 months). With indeterminate LDCT results (NODCAT III), a follow-up scan was performed at 3 months. With positive LDCT results (NODCAT IV), the patient was referred to a pulmonologist.

If the follow-up scan result was negative, GROWCAT A or B, no further procedures were undertaken. With GROWCAT C scan, the patient was referred to the pulmonology department for further evaluation. At the 1-year screening round, negative findings (NODCAT I, GROWCAT A) proceeded to study follow-up phase while with individuals with indeterminate LDCT results (NODCAT II or GROWCAT B), a follow-up scan was performed at 12 months. With new NODCAT III findings, a follow-up scan was performed at 3 months, and with positive LDCT results (NODCAT IV or GROWCAT C), the patient was referred to pulmonologist for further evaluation.

### Approvals

The study was approved by the Ethics committee of Northern Ostrobothnia Hospital District (EETTKM 21/ 2022) and registered at clinicaltrials.gov (NCT05630950). All the participants signed an informed consent before any study procedures. The study subjects were not compensated for their participation. Of note, LDCT lung cancer screening is not among the publicly funded cancer screenings in Finland. The study was conducted in accordance with the Declaration of Helsinki and Good Clinical Practice guidelines.

### Statistical analysis

Data analysis was carried out using SPSS version 29.0.1. For dichotomic variables, Two-sided Pearson Chi-Square test was used to calculate univariate Odds ratios with 95% Wald confidence limits. Results with *p*-values of <0.05 were considered statistically significant.

## Results

### Cohort characteristics

The study included 200 randomized participants. The mean age was around 60 years, with an even gender distribution (female *n* = 102; 51.0%). Most participants were in a relationship (*n* = 142; 71.0%). Prior comorbidities (by ICD-10 diagnosis) were reported in (*n* = 158; 79.0%) screening subjects. Smoking history revealed a median daily consumption of 15 cigarettes, 31 pack-years, and a median score of 3 in three-question Fagerström test. Participants reported a median of two prior smoking cessation attempts. Self-reported asbestos exposure was rare (*n* = 24; 12.0%), but nearly half (*n* = 87; 43.5%) reported exposure to smoke, dust, or fumes. Previous serious infections were reported by 26% (*n* = 24) of the participants. Characteristics are shown in detail in Table 1.

**Table 1:** Demographics.

	*n* (%)/mean
All	200 (100)
Age	60.5
**Gender**	
Male	98 (49.0)
Female	102 (51.0)
**Relationship status**	
Single	58 (29.0)
In a relationship	142 (71.0)
**Co-morbidities**	
No	42 (21.0)
Yes	158 (79.0)
How many cigarettes/day	15.0
Pack years	31.0
Fagerström test	3.0
How many times quitted	2.0
**Self-reported asbestos exposure**	
No	176 (88.0)
Yes	24 (12.0)
**Self-reported exposure to smoke, dust, or fumes**	
No	113 (56.5)
Yes	87 (43.5)
**Previous serious infections**	
No	148 (74.0)
Yes	52 (26.0)

### Recruitment channels

The study used various recruitment sources. We informed all the relevant healthcare units (pulmonary medicine, internal medicine, and surgery) in Oulu University hospital, and local outpatient care (health centers and occupational health) of the region. Furthermore, study information was published in the Oulu University hospital’s internet pages and advertised twice in the leading subscription and free distribution newspaper of the Oulu area. Advertisement included a short description of the main inclusion/exclusion criteria, study procedures, and contact information. Of the recruitment channels, newspaper advertisements were the most effective channels (*n* = 149; 74.5%). Subscription-based newspaper advertisement accounted for 52.% (*n* = 105) and non-subscription newspaper ads 22.0% (*n* = 44). Internet advertisements (*n* = 23; 11.5%) and healthcare referrals (*n* = 5; 2.5%) contributed minimally, with some participants citing other or unknown sources (*n* = 23; 11.5%). Participants over 65 years old were more likely to be recruited by newspaper advertisements (*n* = 69; 34.5%) than internet-based advertisements (*n* = 4; 5.1%). As expected, participants under 65 years old were more likely to be recruited by internet advertisement (*n* = 19; 15.7%) than their older counterparts. (Person Chi-Square *p* = 0.0008) (Table 2).

**Table 2:** Study recruitment channels.

	All *n* (%)	< 65 years *n* (%)	≥ 65 years *n* (%)
All	200 (100)	121 (60.5)	79 (39.5)
Recruitment channel			
Subscription newspaper advertisement	105 (52.5)	57 (47.1)	48 (60.8)
Non-subscription newspaper advertisement	44 (22.0)	23 (19.0)	21 (26.6)
Internet advertisement	23 (11.5)	19 (15.7)	4 (5.1)
Referral from healthcare unit	5 (2.5)	5 (4.1)	0 (0)
Other	14 (7.0)	11 (9.1)	3 (3.8)
Unknown	9 (4.5)	6 (5.0)	3 (3.8)

### LDCT screening results and detected lung cancers

Screening uptake was robust across both screening rounds. All the study participants (*n* = 200) underwent LDCT examination at baseline. The median time to LDCT from randomization was 1.7 weeks (range 0.1 to 7.4 weeks) and 97% (*n* = 196) underwent the LDCT within protocol defined 6 weeks from randomization (not shown). For the second screening round at year 1, 196 individuals were eligible for screening examination. Four individuals were non-eligible because of identification of lung cancer (*n* = 2), other metastatic cancer (*n* = 1), or death (*n* = 1). At year 1, 183 of the eligible 196 (93.4%) participants underwent the LDCT examination (Table 3).

**Table 3:** Screening test results by screening round.

	Screening uptake (%)	Indeterminate test (%)	Positive test (%)	Detection of lung cancer (%)	Positive predictive value (%)
Round 1.	200/200 (100)	14/200 (7.0)	4/200 (2.0)	2/200 (1.0)	50
Round 2.	183/196 (93.4)	4/183 (2.2)	4/183 (2.2)	4/183 (2.2)	100
Total	383/396 (96.7)	18/383 (4.7)	8/383 (2.1)	6/383 (1.6)	75

Detection rates for indeterminate and positive tests were 7% (*n* = 14) and 2% (*n* = 4) in Round 1, respectively, and lung cancer was detected in two individuals (1.0%). At the second screening round, detection rates for indeterminate and positive tests were 2.2% for both (*n* = 4+4), respectively, and lung cancers were detected in four participants (*n* = 4; 2.2%). In total, indeterminate and positive tests were identified in 18 (4.7%) and eight (2.1%) individuals. Six cases of lung cancer were identified in the study, with an excellent cumulative positive predictive value of 75% of LDCT positive findings. LDCT Screening results are shown in detail in Table 3. All identified cancers were non-small cell lung cancer (NSCLC). Most of the detected cancers (*n* = 5; 83.3%) were detected at Stage I while one patient was diagnosed at stage III. Initial treatment included curative surgery in four patients, stereotactic body radiotherapy (SBRT) and palliative radiotherapy for the rest. Disease-free status was maintained during a median follow-up of 8.4 months (range 1.3–16.2 months). Identified lung cancers are shown in detail in Table 4.

**Table 4:** Identified lung cancers and their stage, histology, treatment, and status.

	All *n* (%)/mean (range)
All	6 (100)
Median follow-up time Stage	8.4 months (1.3–16.2)
I	5 (83.3)
II	0 (0)
III	1 (20.0)
IV	0 (0)
**Histology**	
NSCLC	5 (83.3)
Other	0 (0)
Unknown	1 (16.7)
**Initial treatment**	
Curative intent surgery	4 (66.7)
SBRT	1 (16.7)
Palliative radiotherapy	1 (16.7)
Disease relapse/progression	
No	5 (83.3)
Yes	0 (0)
Unknown	1 (16.7)

NSCLC: non-small cell lung cancer; SBRT: stereotactic body radiotherapy.

### Long-term smoking cessation results

Smoking cessation rate was higher in the application arm, with 18.3% of non-smokers (*n* = 17) compared to controls (12.8%; *n* = 12) at 1 year time-point, with a non-significant odds ratio (odds ratio [OR]: 1.5, 95% confidence interval [CI]: 0.7–3.4). In the application arm, smoking cessation rates remained consistently similar at the 3-, 6- and 12-month time points. Notably, participants in the control group were offered access to the smartphone application at 6-month time-point, which may have influenced the smoking cessation results at 1 year. We also carried out an exploratory analysis on individuals who were non-smokers at least for 6-months at the 1-year time point. Results of this analysis favored smartphone application (OR: 2.6, 95% CI: 0.9–7.8) with similar OR compared to 3 or 6-months. Long-term smoking cessation results are shown in Table 5.

**Table 5:** Smoking status at 1 year.

	Application	Control	OR (95% CI)	*p* *
	*n* (%)	*n* (%)		
All	93 (100)	94 (100)		
Smokers	76 (81.7)	82 (87.2)		
Non-smokers	17 (18.3)	12 (12.8)	1.53 (0.69–3.41)	NS
Non-smokers (≥ 6 months)	12 (12.9)	5 (5.3)	2.64 (0.89–7.81)	NS

* Person Chi-Square test.

## Discussion

This study was aimed to investigate the feasibility of LDCT lung cancer screening in Finland along smoking cessation. This pilot study provides valuable insights into hurdles of implementation of LDCT screening for lung cancer in Finland. Our findings suggest that some of the LDCT screening obstacles can be successfully handled within the Finnish healthcare system, with high screening uptake rates, a favorable detection rate for early-stage lung cancer, and consistent long-term smoking cessation outcomes.

Invitations to existing cancer screening programs are based on specific age and gender [14, 15]. Since lung cancer screening is cost-beneficial only in individuals with marked smoking history, and registries of smokers are lacking, novel strategies are needed for recruitment. In general, mass invitation, targeted invitation based on healthcare risk data, referral, or volunteering are established strategies for recruitment to lung cancer screening [16]. One of the main exploratory endpoints of this study included analysis of the recruitment channels, which is relevant for the implementation of LDCT in the Finnish healthcare system. We investigated both referral and volunteering in this study since these were considered as the most feasible strategies if LDCT screening would be adopted. Newspaper advertisements emerged as the most effective recruitment channel, particularly among participants aged 65 and older. In contrast, internet-based advertisements were more effective for younger participants, indicating the need for tailored approaches to reach diverse age groups. Interestingly, only a few referrals were made from healthcare sources, highlighting an area with potential for improvement. Contradictory to our findings, a LDCT screening pilot in Estonia has shown that referrals from family physicians are a very effective method to invite individuals to LDCT screening [17]. It is likely that a hybrid strategy with variable volunteer engagement, referrals, and targeted high-risk identification would lead to the best recruitment results and cost-effectiveness.

High number of false positive findings has been identified as one the most important hurdles of lung cancer screening. NSLT trial investigating LDCT screening using lesion diameter as a method of assessment showed very high (96.5%) false positive rate [3]. NELSON study investigated lesion volume as defining criteria and was able to reduce the number of false positives [2]. Because of the lower false positive rate and better cost-effectiveness, we utilized NELSON lesion protocol in our study [18]. We were able to demonstrate low rates of indeterminate findings, which were associated with minimal risk to lung cancer, and most indeterminate findings were detected at round one. With positive lesions, we observed a high positive predictive value (75%) for lung cancer detection and the results exceeded the one of NELSON trial [2]. Notably, the majority of detected lung cancers were diagnosed at stage I (83%), allowing for curative treatment in most of them. Our study results suggest that the performance of LDCT is good in our research context and LDCT screening can detect marked number of localized cancers, which can be treated with curative intent. Overdiagnosis is considered as a potential harm of all cancer screening which leads to anxiety, physical harm, and costs without benefits. NLST and NELSON trials have shown that with longer follow-up, 3–8.9% of LDCT screening identified lung cancers are overdiagnosed [19].

For a cancer screening to be effective, adherence to screening protocol is crucial. A major challenge in lung cancer screening is patient dropout between screening rounds. In our study, adherence to screening was robust with screening uptake of 96.7%. This is very similar to the uptake of LDCT screening in both NLST and NELSON studies (90–95%) [2, 3]. Lung cancer screening uptake has been low and in US, only 16.4% of the eligible participate in screening with high variance between the states (8.6–28.7%) [20]. However, the screening uptake in US has been increasing from program initiation [21]. This suggests that efforts should be made to increase screening uptake in real-world setting, which also requires time and resources.

Smoking cessation at 3- and 6-months were the primary endpoints of the LDCT-SC-FI study, which have previously been shown to favor the use of Suunta smartphone application for higher abstinence rates [12]. The smartphone application was also offered to the control group at 6-months and therefore, we expected no difference between the study groups at 1-year. However, it is worth noticing that the percentage of non-smokers was similar in the application arm at 1 year compared to 3 and 6-month timepoints, and the rate of long-term non-smokers to be higher in the application arm. Smoking cessation is recommended to be integrated to lung cancer screening program [7–10]. Our study supports the concept of smoking cessation intervention with smartphone application with good short-term and long-term performance.

Our study has some obvious limitations. The study recruitment occurred only in a single cancer center and the number of study participants was low in the context of cancer screening. We only investigated volunteering and referral, as recruitment channels and our study does not bring information on other recruitment methods such as risk-based targeting. Furthermore, recruitment is likely to bare area and country-specific hurdles and our results might not be transferable to other countries. Since the study population was mainly composed of volunteers, this may introduce selection bias in which individuals with health-conscious and in a better socioeconomic position are being overrepresented. Interestingly, our study population had an equal gender distribution that is different from prior lung cancer screening trials, which included mainly males (59–84%) [2, 3]. Some studies have suggested that females might benefit more from screening [2]. Therefore, our study adds more information on the effects of lung cancer screening and smoking cessation in females. Even though double reading of CT scans is recommended in lung cancer screening, we used single reading because of limited radiological resources and the feasibility nature of the lung cancer screening part of the study. Furthermore, smoking cessation was assessed by self-reported measure without biochemical affirmation, which might overestimate the number non-smokers.

## Conclusions

Our study results suggest that lung cancer screening by volunteering is feasible in Finland. LDCT has high uptake rates and good performance measures for lung cancer detection and false positives. Furthermore, smoking cessation with smartphone application is feasible for lung cancer screening program with improved short- and long-term cessation rates.

## Data Availability

Data collected for the study will be made available on a reasonable request to the corresponding author.
